# A case of reversible hypoparathyroidism in a patient with Riedel's thyroiditis treated with glucocorticoids

**DOI:** 10.1002/ccr3.7085

**Published:** 2023-03-15

**Authors:** Salma Salhi, Ibtissem Oueslati, Sabrina Ayari, Elyes Kamoun, Meriem Yazidi, Melika Chihaoui

**Affiliations:** ^1^ Department of Endocrinology, Faculty of Medicine La Rabta University Hospital, University of Tunis‐El Manar Tunis Tunisia

**Keywords:** glucocorticoids, hypocalcemia, hypoparathyroidism, Riedel's thyroiditis

## Abstract

A 48‐year‐old woman with a history of primary hypothyroidism, presented with compressive symptoms secondary to a rapid enlargement of a preexisting goiter. She had no clinical signs of hypocalcemia. Biological tests revealed hypoparathyroidism. Cervicothoracic computed tomography scan showed a heterogeneous compressive goiter. The patient was treated with levothyroxine, calcium, and alfacalcidol. A total thyroidectomy was not performed because of the hard adhesion to neighboring structures. Histopathological examination of the thyroid biopsy was consistent with the diagnosis of Riedel's thyroiditis (RT). The patient was treated with glucocorticoids. The outcome was marked by the resolution of compressive symptoms and the decrease of the thyroid gland volume. Serum calcium and parathyroid hormone levels reached normal ranges after the discontinuation of vitaminocalcic supplementation. Hypoparathyroidism may be clinically asymptomatic in a patient with RT as in our case. Early administration of glucocorticoids may be effective in reducing the fibrosclerotic process and lead to the recovery of parathyroid dysfunction.

## Graphical abstract

Hypoparathyroidism secondary to Riedel's thyroiditis (RT) may be asymptomatic. Glucocorticoids may reverse the fibrosclerotic process and parathyroid dysfunction if introduced early in the disease.

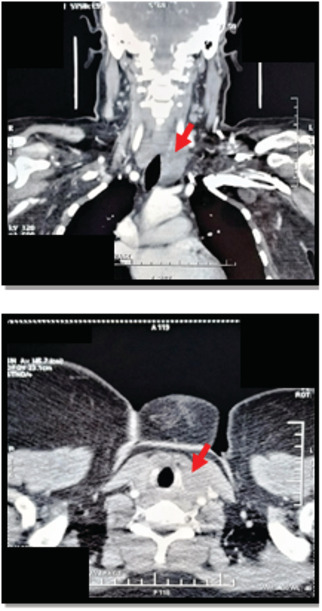

## 1 INTRODUCTION

Riedel's thyroiditis (RT) is a rare benign fibroinflammatory thyroid disease. Its estimated incidence is suggested to be one case per 100,000 inhabitants.^1^ The pathophysiological mechanisms of RT are still unclear. Three possible theories were suggested. The most defended one is the thyroid involvement by the multifocal fibrosclerotic process based on the association of RT with other fibroinflammatory conditions such as retroperitoneal fibrosis, mediastinal fibrosis, and sclerosing cholangitis.^2^ Other clinical, serologic, and histological features support the autoimmune origin.^3^, ^4^ Finally, RT could be a part of a systemic disease associated with IgG4‐positive plasmocytes.^4^, ^5^

RT may invade adjacent tissues including the parathyroid glands, skeletal muscles, nerves, and blood vessels, as well as the trachea. The involvement of the parathyroid glands was reported in 14% of patients with RT.^6^

Herein, we report a case of asymptomatic hypoparathyroidism in a patient with RT which was reversible with effective administration of glucocorticoids.

## 2 CASE PRESENTATION

A 48‐year‐old woman was referred to our department for a compressive goiter. Her past medical history included primary hypothyroidism diagnosed at the age of 46 years and treated with 100 μg per day of levothyroxine. Her initial cervical ultrasound showed a heterogeneous goiter with a right lobar nodule of 22 mm and a left lobar nodule of 18 mm. Thyroid peroxidase antibodies were not available. There was no history of radiation exposure or family history of thyroid cancer.

She presented with rapid enlargement of the preexisting goiter with dysphagia and dyspnea. She had no chronic diarrhea, no flushes, and no symptoms of hypocalcemia.

On physical examination, she had a body weight of 119 kg, a height of 1.63 m corresponding to a body mass index of 44.8 kg/m^2^, a body temperature of 37.2°C, a blood pressure of 125/80 mmHg, a heart rate of 90 beats/min, a painless heterogeneous multinodular goiter with hard consistency and non‐palpable lower poles.

The results of biological and hormonal investigations are shown in Table 1. Biological tests showed a total corrected calcemia of 1.95 mmol/L with a low PTH level, consistent with the diagnosis of hypocalcemia secondary to hypoparathyroidism.

**TABLE 1:** Biological and hormonal parameters.

	Values	Reference ranges
Total‐serum calcium (mmol/L)	1.87	2.14–2.54
Albumin (g/L)	35.7	40.2–47.6
Corrected‐calcium^a^ (mmol/L)	1.95	
Serum phosphorus (mmol/L)	1.25	0.87–1.45
Calcium to phosphorus ratio	1.57	
Intact‐PTH (ng/L)	12	15–68
Fasting blood glucose (mmol/L)	5.28	4.1–6
Total‐cholesterol (mmol/L)	4.6	3.1–5.17
Triglycerides (mmol/L)	2.58	1.29–5.17
HDL‐cholesterol (mmol/L)	0.8	0.9–1.81
Natremia/kalemia (mmol/L)	137/3.7	135–145/3.6–5.0
Serum creatinine (μmol/L)	61.9	35.3–114.9
Serum magnesium (mmol/L)	0.82	0.65–1.00
White blood cells/mm^3^	7810	4000–10,000
Lymphocytes/mm^3^	3100	1500–4000
Neutrophils/mm^3^	3820	1500–7000
Hemoglobin (g/dL)	12.8	12–16
CRP (mg/L)	15.6	3–5
TSH (mIU/L)	0.39	0.35–4.95
FT4 (ng/dL)	1.2	0.71–1.85

^a^ The albumin‐corrected calcemia was calculated according to the following formula: total calcemia (mmol/L) + 0.02 × (40‐ albuminemia (g/L)).

Antithyroid peroxidase antibodies were negative.

**FIGURE 1:**
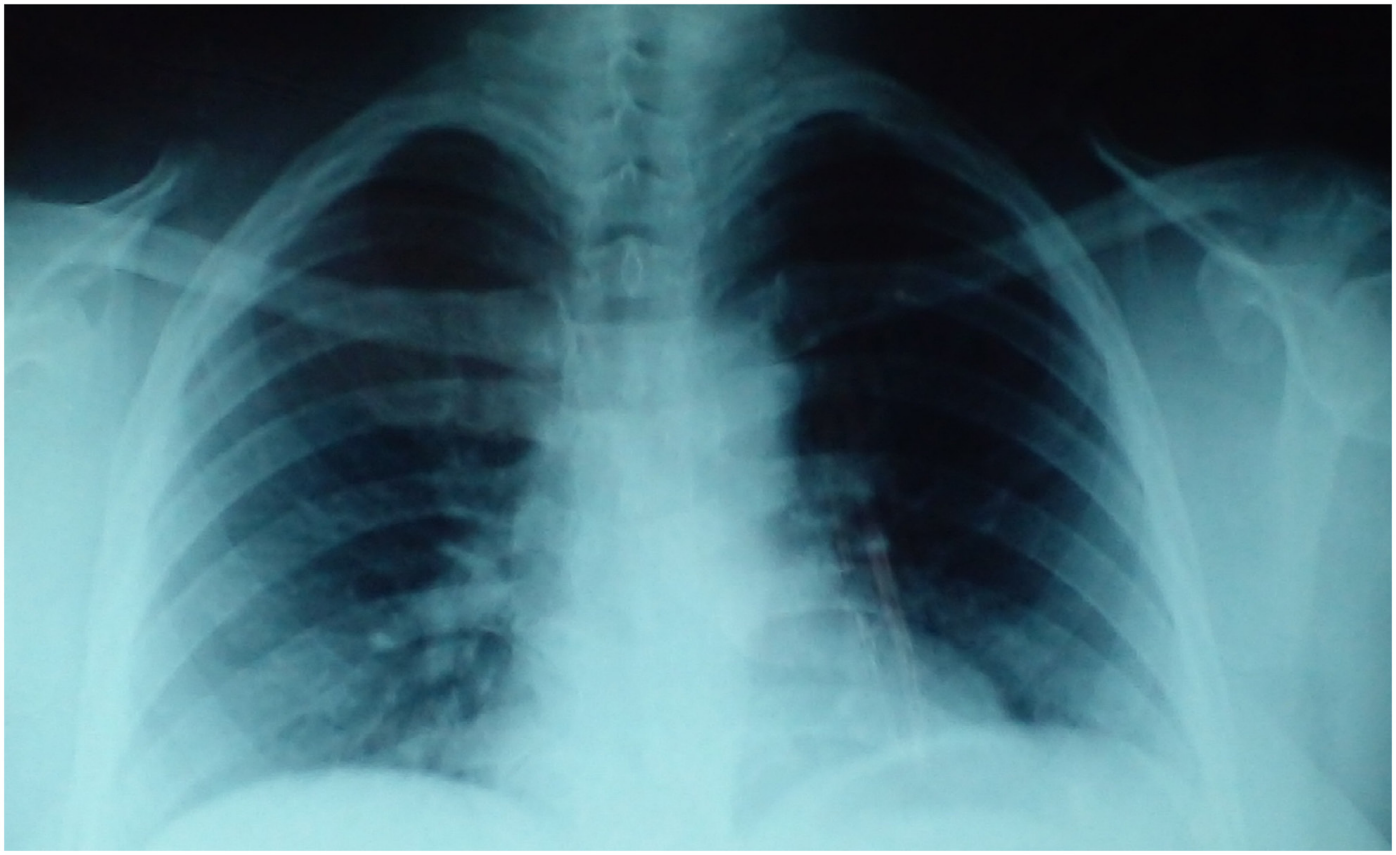
Chest X‐ray showing a plunging and compressive goiter.

**FIGURE 2:**
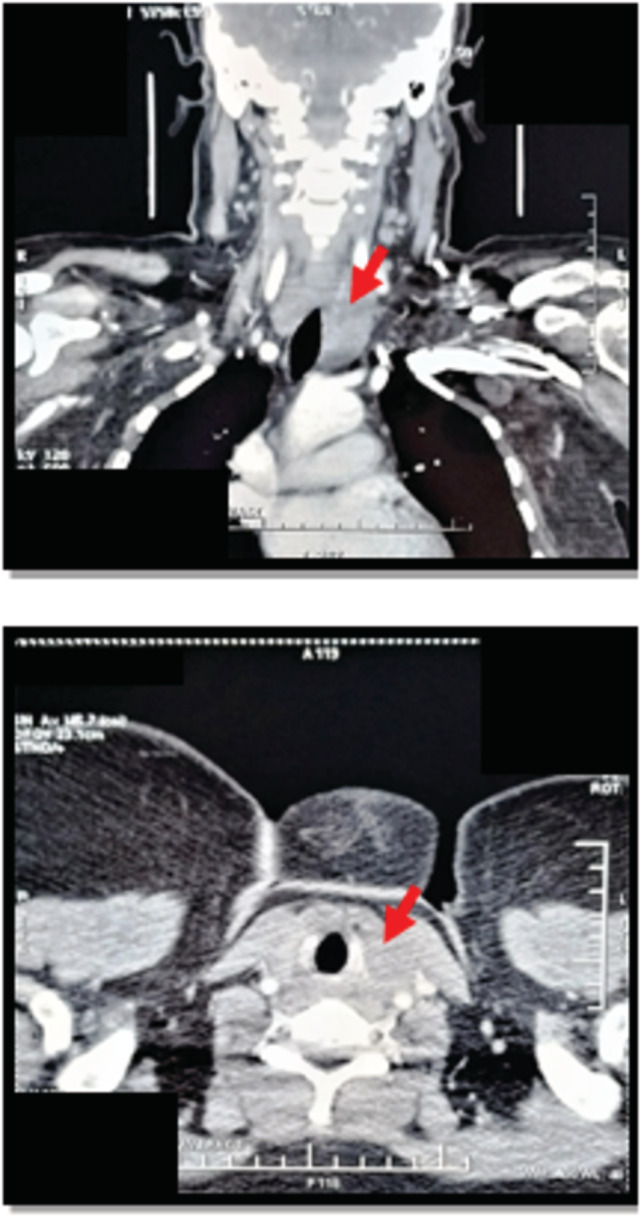
Cervico‐thoracic computed tomography scan showing the left thyroid lobe plunging into the mediastinum.

Chest X‐ray showed a plunging and compressive goiter (Figure 1). Thyroid ultrasound showed enlarged thyroid lobes, the right measuring 53 × 20 × 27 mm and the left 59 × 29 × 36 mm, and three isoechoic nodules measuring 17, 18, and 23 mm, with macrocalcifications. Cervicothoracic computed tomography scan showed a heterogeneous goiter with the left lobe plunging into the mediastinum compressing the left internal jugular vein, esophagus, and trachea (Figure 2). There was no cervical or mediastinal adenopathy. The patient was treated with levothyroxine (100 μg/day), calcium (1500 mg/day), and alfacalcidol (1 μg/day). A total thyroidectomy was indicated. Surgical findings depicted the presence of a large thyroid tumor of stony‐hard consistency with infiltration of the trachea and muscles. It was not possible to dissect the tumor because of the hard adhesion to neighboring structures. Thus, only decompression and biopsy were performed. The histopathological examination showed a fibrous tissue with a diffuse inflammatory infiltrate composed of lymphocytes, plasma cells, and rare neutrophils with no signs of malignancy, consistent with the diagnosis of RT. The patient was treated with prednisone at the dose of 20 mg/day for 12 months. The outcome was marked by the resolution of compressive symptoms and the diminution of the thyroid gland volume. Thus, the corticosteroid dose was gradually reduced. No side effects were observed. The patient was regularly followed up at the outpatient department. Serum calcium and parathyroid hormone levels reached normal ranges after the discontinuation of vitaminocalcic supplementation (total serum calcium level: 2.22 mmol/L and intact‐PTH level: 65.7 ng/L). The thyroid ultrasound showed a normal‐sized hypoechoic thyroid gland with loss of its usual structure, a solid right nodule of 8 mm, and the absence of invasion of adjacent tissues.

## 3 DISCUSSION

Riedel's thyroiditis is a rare disease that affects women aged between 30 and 50 years.^7^ Its clinical presentation and diagnosis are often complex. The most common symptoms in patients with RT are thyroid enlargement (89%), dyspnea (50%), hoarse voice (41%), and neck pain (41%).^1^ Other signs such as exophthalmos and venous sinus thrombosis were reported. Symptoms develop in a few months; generally in a median of 4 months.^1^ Our patient presented with rapid enlargement of a preexisting goiter with compressive symptoms.

In patients with RT, physical examination shows a hard goiter, which may be painful and immobile due to its adherence to the adjacent tissues.^6^

The imaging findings may contribute to the diagnosis of RT and help to determine the extension of the fibrosis to the laterocervical tissues. The thyroid tissue appears homogeneously hypoechoic on ultrasound and hypodense‐to‐normal on CT scan.^8^ On magnetic resonance imaging, the thyroid was reported to be hypointense on both T1‐ and T2‐weighted images.^8^

Fine‐needle aspiration biopsy is not helpful in the diagnosis of RT with non‐conclusive results in 75% of cases.^2^ Therefore, RT is usually diagnosed through a surgical biopsy in 82% of cases.^1^ Its typical histological findings are atrophy and inflammation of the thyroid parenchyma with dense fibrosis extending to the adjacent tissue.

The main differential diagnosis of RT is thyroid malignancy, especially anaplastic thyroid carcinoma, thyroid lymphoma, and sarcoma. In some cases, RT may be difficult to differentiate from the fibrosing form of Hashimoto's thyroiditis. The fibrosing variant of Hashimoto's thyroiditis is rare, occurring in about 10% of cases, and is characterized by an extensive replacement of the thyroid parenchyma by fibrosis without extension into the surrounding structures.^9^ Moreover, the coexistence of Hashimoto's thyroiditis and RT in the same patient was reported in the literature.^9^, ^10^

Thyroid function depends on the extent of fibrosis which may replace the normal thyroid tissue. In the majority of cases, RT is associated with normal thyroid function. Hypothyroidism was reported in 30% of cases.^11^ It results from thyrocyte destruction. In 46% of cases, hypothyroidism was present before the diagnosis of RT as in our patient.^1^ Thyroid antibodies were positive in 50% of cases.^1^ In our patient, thyroid antibodies were not available at the diagnosis of hypothyroidism and were negative at the diagnosis of RT. Inflammatory markers such as C‐reactive protein are slightly elevated in 72% of patients as in our case.^1^

Adjacent tissues, in particular the parathyroid glands and muscles, may be involved by fibrosis in patients with RT. Hypoparathyroidism is rare.^4^, ^12^, ^13^ It may result from the compression or destruction of the parathyroid glands by the invasive fibrosclerotic process.^2^ It was reported in 14% of patients with extracervical fibrotic processes. Other case reports described primary hypoparathyroidism preceding RT.^14^ In our case, there were no clinical manifestations of hypocalcemia reflecting the slow decrease of serum calcium levels. Biochemical data confirmed the diagnosis of hypoparathyroidism and the patient was treated with vitamin D and calcium. Patients may present with fibrosis in variant distant sites of the body. Two‐thirds of patients with RT will not develop extracervical fibrosis within 10 years of evolution.^15^ Fatourechi et al.^2^ reported a fibrosing mediastinitis with the diagnosis of RT in three patients and 10 years later in one case. Orbital fibrosis, pancreatic fibrosis, and epidural space fibrosis were also reported.

Due to the rarity of RT, there is no consensus regarding the treatment and follow‐up of this disease. Surgical treatment is indicated in patients with compressive symptoms. However, the complete removal of the thyroid gland may be difficult due to deep adjacent tissue infiltration and the risk of complications such as hypoparathyroidism and recurrent nerve damage.^2^ Thyroid isthmectomy can be performed to relieve the obstruction.

Medical treatment with glucocorticoids is often prescribed in patients with RT. Prednisone is the most commonly used molecule. The dose and duration of the therapy depend on the response and tolerance The average daily dose is 15–60 mg and the median duration of treatment is about 3 months in most cases.^1^, ^3^, ^16^ In case of a life‐threatening dyspnea, prednisolone can be administered parenterally at the dose of 2–3 mg/kg/day for 2–7 days according to the clinical response.^16^ Glucocorticoids are more effective if introduced early in the disease.^16^ Our patient was treated with prednisone at the dose of 20 mg/day for 12 months. The outcome was marked by the resolution of compressive symptoms and the diminution of the thyroid gland volume with no side effects.

Tamoxifen can be used as a second agent in the treatment of RT. It inhibits fibroblast proliferation and collagen production in the thyroid tissue.^17^ The prescribed dose is 10–20 mg twice a day for a mean duration of less than a year. Tamoxifen was an effective and well‐tolerated treatment either in combination with glucocorticoids or as monotherapy.^18^, ^19^, ^20^, ^21^ However, it should be used with caution in patients with predisposing factors for deep vein thrombosis.^19^

In the meta‐analysis of Zala et al.,^1^ 90% of cases had a good prognosis with improvement or resolution of their symptoms following treatment. Untreated RT is usually slowly progressive, although it may stabilize or even regress spontaneously in some cases.

In our case, there were an improvement of the compressive symptoms, a total recovery of hypoparathyroidism, and a gradual reduction of the goiter volume. Hypoparathyroidism reversibility has already been reported in a 40‐year‐old woman with RT who had a thyroid isthmectomy and was treated with corticosteroids and tamoxifen.^22^ In the majority of reported cases, the parathyroid dysfunction was permanent requiring continued supplementation with calcium and active vitamin D.

## 4 CONCLUSION

RT is a rare thyroid disease with extending fibrosis to surrounding tissues. The involvement of the parathyroid glands may be clinically silent. Early surgical decompression of the goiter and the administration of glucocorticoids may be effective in reducing the fibrosclerotic process and lead to the recovery of parathyroid dysfunction. However, in the majority of cases, hypoparathyroidism is permanent requiring continued treatment.

## AUTHOR CONTRIBUTIONS

**Salma Salhi:** Data curation; formal analysis; resources; writing – original draft; writing – review and editing. **Ibtissem Oueslati:** Conceptualization; data curation; formal analysis; investigation; methodology; project administration; supervision; validation; visualization; writing – original draft; writing – review and editing. **Sabrina Ayari:** Data curation; formal analysis; validation; writing – review and editing. **Elyes Kamoun:** Data curation; formal analysis; validation; writing – review and editing. **Meriem Yazidi:** Data curation; formal analysis; investigation; validation; visualization; writing – review and editing. **Melika Chihaoui:** Data curation; formal analysis; investigation; supervision; validation; visualization; writing – review and editing.

## FUNDING INFORMATION

None.

## CONFLICT OF INTEREST STATEMENT

The authors declare that they have no conflicts of interest.

## ETHICS STATEMENT

Ethical approval for this case report was not required.

## CONSENT

Written informed consent was obtained from the patient for the publication of this report.

## Data Availability

Data sharing is not applicable – no new data is generated.
